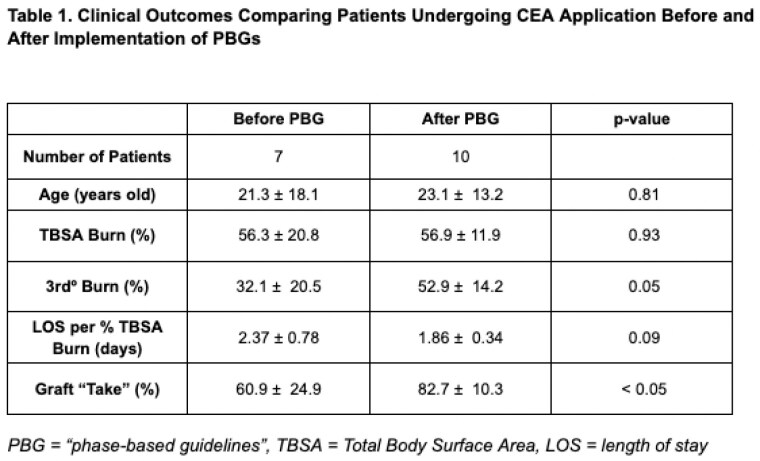# 559 Where Are We Now? Cultured Epithelial Autograft Application and Phase-Based Guidelines for Massive Burns

**DOI:** 10.1093/jbcr/iraf019.188

**Published:** 2025-04-01

**Authors:** Megan Wojick, Kathleen Ewanowski, Irma Fleming, Giavonni Lewis, Christopher LaChapelle, Callie Thompson

**Affiliations:** University of Utah Health; University of Utah Health Burn Center; University of Utah Burn Critical Care; University of Utah Health; University of Utah Health; University of Utah Health

## Abstract

**Introduction:**

Although cultured epithelial autografts (CEA) have been clinically utilized for over 40 years, CEA remains available mostly for compassionate use. CEA use has been associated with lower mortality in massive-burn patients, however, CEA patients are more susceptible to bacterial contamination and complete graft loss than traditional split-thickness autografts. In an effort to help minimize infection and maximize graft “take”, our institution implemented standardized “phase-based guidelines” (PBGs) for patient care in 2020. In this review, we aimed to report our clinical outcomes in the four years since implementation.

**Methods:**

A retrospective chart review was performed on all patients who underwent application of CEA in our burn center from 2018-2024. Data extraction included demographics, percent TBSA burn, percent 3rd degree burn, number of surgeries, number of CEA applications, percent “take” after each CEA procedure, need for regrafting, length of stay (LOS), and mortality. Photographs were used retrospectively to determine percent grafted with CEA, percent “take” at 30 days post-op, and percent TBSA re-grafted for each patient.

**Results:**

Seventeen patients met study inclusion criteria. Seven patients underwent CEA application prior to implementation of PBGs while ten had PBGs used in their care. There was no significant difference in average age or overall % TBSA of the patients in both groups. There was a statistically significant difference in percent “take” at 30 days post-op following CEA application with an average of 60.9% take for patients who did not have PBGs and 82.7% take for patients who did. All ten patients in the PBG group had a biodegradable temporizing matrix (BTM) prior to CEA application while it was only used in three patients in the pre-PBG group. Two patients in each group required re-grafting with on average 13.6 % TBSA per surgery in the pre-PBG group and 9.1 % TBSA per surgery after PBG use. The LOS per %TBSA for patients prior to PBGs was 2.37 days and 1.86 days after implementation (p=0.085).

**Conclusions:**

There has been an overall improvement in clinical outcomes of patients undergoing CEA application at our institution following the implementation of PBGs, specifically in average % “take” of CEA. There has also been a clinically significant difference in LOS per %TBSA between the groups which allows for an earlier discharge from the hospital for these patients.

**Applicability of Research to Practice:**

PBGs can be scaled and implemented at other burn centers to improve CEA application outcomes.

**Funding for the Study:**

N/A. This research did not receive any specific grant from funding agencies in the public, commercial, or not-for-profit sectors.